# The relationship between elderly suicide rates and different components of education: a cross-national study

**DOI:** 10.5249/jivr.v4i2.75

**Published:** 2012-07

**Authors:** Ajit shah

**Affiliations:** ^a^University of Central Lancashire, Preston, United Kingdom and Consultant Psychiatrist, West London Mental Health NHS Trust, London, United Kingdom.

## Abstract

**Background:**

Two recent studies reported a curvilinear (U-shaped) between elderly suicide rates and educational attainment measured by the United Nation’s Education Index. A study examining the curvilinear (U-shaped) relationship between elderly suicide rates and the individual components of the Education Index (adult literacy rate, percentage of children of relevant age group enrolled in primary schools and percentage of children of relevant age group enrolled for secondary schools) and one other measure of educational attainment (youth literacy rate) was undertaken to partial out the effects of the individual components of Education Index on elderly suicides.

**Methods:**

A cross-national study examining the relationship between elderly suicide rates (Y-axis) and different measures of educational attainment (X-axis) was undertaken using data from the World Health Organization and the United Nations data banks using Curve estimation regression models.

**Results:**

The relationship between elderly suicide rates with the adult literacy rate, the percentage of children of relevant age group enrolled for secondary schools and the youth literacy rate was curvilinear (U-shaped curve). This relationship was absent with the percentage of children of relevant age group enrolled in primary schools.

**Conclusion:**

Given the cross-sectional study design, a causal relationship between elderly suicide rates and measures of educational attainment, including the adult literacy rate, the percentage of children of relevant age group enrolled for secondary schools and the youth literacy rate, cannot be assumed. However, the findings suggest that future studies of elderly suicide rates and educational attainment should focus on the adult literacy rate, the percentage of children of relevant age group enrolled for secondary schools and the youth literacy rate as measures of educational attainment.

## Introduction

The elderly population size is increasing in most countries^1^Traditionally, suicide rates increased with ageing. A recent cross-national study of 62 developing and developed countries reported an increase in suicide rates with ageing in males and females in 25 and 27 countries respectively.^3^Thus, suicides in the elderly are an important public health concern. Comprehensive understanding of the substantial worldwide variation in population patterns of suicide may be critical for developing prevention programmes.^4^Detailed knowledge of distil risk and protective factors may also have greater public health relevance for universal prevention strategies.^3,5^

Suicide victims under the age of 25 years lacked educational qualifications.^6^African American suicide victims, of mixed-age groups, had lower levels of educational attainment,^7^particularly those aged 25-54 years.^8^ However, other studies of African American suicide victims either did not observe this association^9^or observed an association with higher levels of educational attainment,^10^particularly in those aged 55+ years.^8^Caucasian suicide victims of mixed-age groups, in the US, had both higher^9^and lower^10^levels of educational attainment. Case-control studies of elderly suicide victims have reported both an association with lower educational attainment^11^and a lack of such an association.^12^

Ecological studies between different countries and between different regions within a country have examined correlations between general population suicide rates and educational attainment. General population suicide rates in Japan,^13^Lithuania,^14^and men in eight European countries^15^were negatively correlated with educational attainment. Moreover, general population suicide rates in the US were observed to have no correlation,^16^ positive correlation^17^and negative correlation^18^with educational attainment; the latter observation may have been an artifact due to inter-state migration amongst the US adult population.^19^Furthermore, general population suicide rates among African Americans^20^and in India^21^ positively correlated with educational attainment. A Canadian ecological study observed a negative correlation between educational attainment and suicide rates in elderly females,^22^but acknowledged that a non-linear relationship between elderly suicide rates and educational attainment may have been missed.^22^

A recent study reported that there is a curvilinear (U-shaped) between elderly suicide rates and educational attainment following a quadratic equation Y = A + BX + CX^2^(where A, B and C are constants, Y is elderly suicide rate and X is educational attainment).^23^A further recent study also replicated this curvilinear relationship using an annual average of five years data on elderly suicide rates and a more recent data set than the earlier study.^24^There are several potential explanations for this curvilinear relationship.^23^First, lower educational attainment may lead to mental illness (and, in turn, suicide) because of neuro-developmental problems, reduced ability to compete for jobs resulting in low income and status, poor problem solving abilities in difficult circumstances and development of anti-social behaviour.^25,26^Second, higher educational attainment may lead to mental illness (and, in turn, suicide), if there is a mismatch between the levels of intelligence and educational attainment and the anticipated socio-economic benefits linked to levels of intelligence and educational attainment, including better jobs, higher income and better housing.^10,27^

The curvilinear relationship between elderly suicide rates and educational attainment was derived using the Education Index, published by the United Nations Development Programme, as a measure of educational attainment.^23,24^The Education Index is a composite measure of the adult literacy rate, and the combined gross enrolment ratios for primary, secondary and tertiary schools. It is possible that one or more components of the Education Index may be important in this curvilinear relationship. Thus, a study examining the curvilinear (U-shaped) relationship between elderly suicide rates and the individual components of the Education Index (adult literacy rate, percentage of children of relevant age group enrolled in primary schools and percentage of children of relevant age group enrolled for secondary schools) and one other measure of educational attainment (youth literacy rate – for those age 15-24 years) was undertaken to partial out the effects of the individual components of Education Index on elderly suicides.

## Methods

Data on elderly suicide rates for males and females in the age-bands 65-74 years and 75+ years for the latest five years were ascertained from the World Health Organization (WHO) (www.apps.who.int/whosis/ database/mort/table1.cfm). The median (range) of the latest available year for the 83 different countries was 2005 (1983-2007); this data set was more recent than that used in an earlier study.^23^An annual average of five consecutive years data on elderly suicide rates was used to minimize year on year random fluctuations in suicide rates.^28^Furthermore a more recent data set then the original study was used.

Data on the components of Education Index (adult literacy rate, percentage of children of relevant age group enrolled in primary schools and percentage of children of relevant age group enrolled for secondary schools) and the youth literacy rates were obtained from the United Nations Development Program (http://hdr. undp.org/en/media/HDI_2008_EN_Tables.pdf). These data were for the year 2004.

Curve estimation regression model was used to test the possible curvilinear relationship (U-shaped curve) between suicide rates and the adult literacy rate, the percentage of children of relevant age group enrolled in primary schools, the percentage of children of relevant age group enrolled for secondary schools and the youth literacy rate fitting the quadratic equation Y = A + BX + CX^2^ (where Y is the suicide rate for elderly, X is adult literacy rate or percentage of children of relevant age group enrolled in primary schools or percentage of children of relevant age group enrolled for secondary schools or youth literacy rates, and A, B, and C are constants). The reason curve estimation regression model was used was that previous studies have used this approach with the education index.

## Results

Table 1 provides characteristics of all the variables. The characteristics of curve estimation regression models are illustrated in Table 2. The relationship between suicide rates for both sexes in both the elderly age-bands with the adult literacy rate, the percentage of children of relevant age group enrolled for secondary schools and the youth literacy rate was curvilinear (U-shaped curve) and fitted the above quadratic equation. This relationship was absent with the percentage of children of relevant age group enrolled in primary schools.

**Table:** Table 1:**Characteristics of all variables studied**

Variable	Mean	Standard Deviation
Suicide Rate Males 65-74 years	24.33	22.52
Suicide Rate Males 75+ years	35.36	31.13
Suicide Rate Females 65-74 years	6.23	6.28
Suicide Rate Females 75+ years	9.05	10.92
Adult Literacy Rate (%)	93.8	6.9
Youth Literacy Rate (%)	97.85	3.45
Primary School Enrolment (%)	94.28	4.48
Secondary Scholl Enrolment (%)	80.96	14.24

Suicide rates are per 100,000 relevant age and gender group. % for education variables measured for relevant age groups

**Table:** Table 2:**Curve estimation regression models for the relationship between elderly suicides and measures of educational attainment**

	R2	Degrees of	F	Significance	Regression Equation
Adult literacy rate (N=45)					
Males 65-74 years	0.33	42	10.38	P<0.0001	Y = 780.75 – 19.26X + 0.12X2
Males 75+ years	0.26	42	7.48	P=0.002	Y = 511.92 – 13.27X +0.09X2
Females 65-74 years	0.30	42	9.09	P=0.001	Y = 154.87 – 3.86X +0.024X2
Females 75+ years	0.38	42	12.64	P<0.0001	Y = 281.43 – 7.04X + 0.044X2
Primary school enrollment (N=71)					
Males 65-74 years	0.01	68	0.34	NS	Y = 673.60 – 13.61X + 0.07X2
Males 75+ years	0.01	68	0.23	NS	Y = 806.57 – 17.04X + 0.09X2
Females 65-74 years	0.05	68	1.80	NS	Y = 620.69 – 13.29X + 0.07X2
Females 75+ years	0.02	68	0.71	NS	Y = 542.06 – 11.72X + 0.06X2
Secondary school enrollment (N=68)					
Males 65-74 years	0.17	65	6.53	P=0.001	Y = 15.56 – 0.58X + 0.008X2
Males 75+ years	0.12	65	4.43	P=0.016	Y = -0.25 – 0.016X + 0.006X2
Females 65-74 years	0.14	65	5.40	P=0.007	Y = -8.60 – 0.19X + 4.69e-5X2
Females 75+ years	0.09	65	3.12	P=0.051	Y = -15.40 – 0.38X + 0X2
Youth literacy rate (N=45)					
Males 65-74 years	0.16	41	4.01	P=0.026	Y = 2699.11 – 60.47X + 0.34X2
Males 75+ years	0.16	41	3.97	P=0.027	Y = 2688.99 – 60.81X + 0.34X2
Females 65-74 years	0.15	41	3.57	P=0.037	Y = 471.51 – 10.71X + 0.061X2
Females 75+ years	0.22	41	5.69	P=0.007	Y = 1055.39 – 23.83X + 0.13X2

Y = Suicide rates X = A measure of educational attainment A, B and C are constants NS= Not significant N = The number of countries with the full data set of suicide rates and the relevant education variable (data on the education variable were not available for all 83 countries.

## Discussion

Some methodological issues need consideration. Cross-national data on suicide rates should be viewed with caution because: data are not available from all countries;^29,30^the validity of this data was unclear;^30,31^ the legal criteria for the proof of suicide vary between countries and in different regions within a country;^30,32^some countries may have poor death registration facilities;^32^and, cultural and religious factors and stigma attached to suicide may lead to under-reporting of suicides.^30,33^Data on the adult literacy rate, the percentage of children of relevant age group enrolled in primary schools, the percentage of children of relevant age group enrolled for secondary schools and the youth literacy rate should also be viewed with caution because: the validity of this data is unclear; and, some countries may have poor registration facilities and infrastructure for collecting educational data.^23^However, data were gathered from the WHO and the UN data bank and was the best and the latest available data set.

There was a curvilinear relationship between suicide rates in both sexes in both the elderly age-bands and the adult literacy rate, the percentage of children of relevant age group enrolled for secondary schools and the youth literacy rate; but this was absent with the percentage of children in the relevant age group enrolled for primary schools. The findings suggest that two of the components of the Education Index (adult literacy rate and percentage of children of relevant age group enrolled for secondary schools) have the same curvilinear relationship as the Education Index. The third component, the percentage of children in the relevant age group enrolled for primary schools, does not demonstrate this curvilinear relationship. This may be an artifact of the methodological issues described above. Alternatively, enrolment in primary schools may not necessarily mean that children attend school or receive appropriate education and thus may not have an association with suicide rates. Also, current figures for enrolment in primary education may be less relevant to elderly suicide rates as the enrolment rates for primary schools may have been very different when the current cohort of elderly people were of primary schooling age. The findings suggest that future studies of elderly suicide rates and educational attainment should focus on the adult literacy rate, the percentage of children of relevant age group enrolled for secondary schools and the youth literacy rate as measures of educational attainment.

The observed curvilinear relationship between elderly suicide rates and the adult literacy rate, the percentage of children of relevant age group enrolled for secondary schools and the youth literacy rate as measures of educational attainment is also consistent with the previously reported relationship between both high and low levels of educational attainment and suicide rates in individual-level and aggregate-level studies (as reviewed in the Introduction). This curvilinear relationship may have several explainations.^23^ First, lower educational attainment may predispose to mental illness (and, in turn, suicide) because of neuro-developmental problems, reduced ability to compete for jobs resulting in low income and status, poor problem solving abilities in difficult circumstances and development of anti-social behaviour.^25,26^Second, higher educational attainment may predispose to mental illness (and, in turn, suicide), if there is a mismatch between the levels of intelligence and educational attainment and the anticipated socio-economic benefits linked to levels of intelligence and educational attainment, including better jobs, higher income and better housing.^10,27^The effects of higher educational attainment may be sustained into old age; this is supported by the association between suicides in male African Americans aged 55+ years and higher levels of educational attainment.^8^

However, given the cross-sectional study design, a causal relationship cannot be assumed. Other potential mechanisms also require consideration. Educational attainment, as measured by the adult literacy rate, the percentage of children of relevant age group enrolled for secondary schools and the youth literacy rate may interact with other factors, may mediate the effects of other factors or their effects may be mediated by other factors. These factors include other protective and risk factors for suicide,^34^genetic factors,^3,5^socio-economic factors,^34,35-37^intelligence,^25^cultural factors,^10,27,36^religious factors^37^and the effect of age, period and cohort.^38,39^The interaction between measures of educational attainment and these factors requires further study as this has important public health implications. If the role of education as a risk factor for elderly suicides can be unequivocally established then: at public health level, policy makers need to ensure that education is made available to all; and at a clinical level, clinicians need to be aware of higher suicide risk in those with low and high levels of education.
